# Mutations in modified virus Ankara protein 183 render it a non-functional counterpart of B14, an inhibitor of nuclear factor *κ*B activation

**DOI:** 10.1099/vir.0.022343-0

**Published:** 2010-09

**Authors:** Laura E. McCoy, Aodhnait S. Fahy, Ron A.-J. Chen, Geoffrey L. Smith

**Affiliations:** Department of Virology, Faculty of Medicine, Imperial College London, Norfolk Place, London W2 1PG, UK

## Abstract

Vaccinia virus (VACV) encodes multiple proteins to evade host innate immunity, including B14, a virulence factor that binds to the inhibitor of *κ*B kinase *β* (IKK*β*) and blocks nuclear factor *κ*B (NF-*κ*B) activation. B14 shares 95 % amino acid identity with the 183 protein encoded by modified virus Ankara (MVA), an attenuated VACV strain being developed as a vaccine vector. To evaluate whether the immunogenicity of MVA might be increased by manipulation of MVA immunomodulatory proteins, the MVA counterpart of B14, protein 183, was characterized. Unlike B14, protein 183 was unstable in eukaryotic cells unless proteasome-mediated protein degradation was inhibited. Furthermore, 183 did not inhibit NF-*κ*B activation in response to cytokine stimulation, and did not restore the virulence of VACV strain Western Reserve lacking gene *B14R*. The instability and non-functionality of 183 are probably explained by a deletion of 6 aa within *α*-helix 6 of the B14 crystal structure.

*Vaccinia virus* (VACV) is the best-studied member of the family *Poxviridae*; it is a double-stranded DNA virus that replicates in the cytoplasm of host cells (Moss, 2007). VACV was used as the vaccine to eradicate smallpox (Fenner *et al.*, 1988) and thereafter has continued to be studied due to its development as a vaccine for other infectious diseases (Smith *et al.*, 1983), and because it is an excellent model for studying virus–host interactions. Although immunoprophylaxis with VACV led to eradication of smallpox, VACV had an imperfect safety record (Lane *et al.*, 1969) and it was recognized that the safety profile needed improvement if VACV was to be used again widely for human vaccination. One strategy to improve safety was to passage VACV strains repeatedly in cell culture until attenuation was achieved, the classical manner in which live-attenuated vaccines were developed for yellow fever, polio, measles, mumps and rubella. In this way, the LC16m8 strain was derived from VACV Lister in Japan (Hashizume *et al.*, 1985), and modified virus Ankara (MVA) was derived from VACV chorioallantois virus Ankara (CVA) in Germany by more than 570 passages in chicken embryo fibroblasts (CEFs) (Mayr & Munz, 1964; Stickl *et al.*, 1974; Mayr *et al.*, 1978). During this serial passage, MVA incurred six major deletions (Meyer *et al.*, 1991) and many smaller mutations (Antoine *et al.*, 1998) compared with the parental CVA (Meisinger-Henschel *et al.*, 2007), and developed a severe restriction in host range so that it is unable to replicate in most mammalian cells (Meyer *et al.*, 1991; Sutter & Moss, 1992). In the later stages of the smallpox-eradication campaign, MVA was used as a smallpox vaccine in over 120 000 individuals without complications (Stickl *et al.*, 1974; Mayr *et al.*, 1978; Mahnel & Mayr, 1994). MVA lacks many immunomodulators that are present in other strains of VACV (Antoine *et al.*, 1998; Blanchard *et al.*, 1998), but other immunomodulators remain and deletion of two of these, B15 and A41, enhanced MVA immunogenicity (Staib *et al.*, 2005; Clark *et al.*, 2006). This paper concerns MVA protein 183, the counterpart of VACV strain Western Reserve (WR) protein B14 (Chen *et al.*, 2006).

VACV WR protein B14 is expressed early after infection in the cytoplasm and is non-essential for virus replication in cell culture (Chen *et al.*, 2006). However, a *B14R* deletion mutant (vΔB14) was attenuated in a mouse intradermal (i.d.) infection model and affected the inflammatory response to infection (Chen *et al.*, 2006). Further study demonstrated that B14 inhibits nuclear factor *κ*B (NF-*κ*B) activation by binding to the inhibitor of *κ*B kinase (IKK) complex via the IKK*β* subunit (Chen *et al.*, 2008). The crystal structure of B14 was solved and revealed a B-cell lymphoma (Bcl)-2 protein fold, despite B14 lacking sequence similarity to Bcl-2 proteins, a family of proteins that regulate apoptosis. Anti-apoptotic Bcl-2 proteins contain a surface groove that binds the BH3 peptide of pro-apoptotic Bcl-2 family members and thereby neutralizes pro-apoptotic activity. In comparison, B14 lacked a surface groove and was not anti-apoptotic (Graham *et al.*, 2008). Given that deletion of *B14R* from VACV WR increased the recruitment of leukocytes to the site of infection (Chen *et al.*, 2006), it was hypothesized that deletion of gene *183R* might increase MVA immunogenicity and increase its potency as a vaccine.

B14 and 183 share 95 % amino acid identity, and there are three sequence differences (Fig. 1a). First, in B14 *α*-helix 1 (*α*1; Fig. 1b), there is a conservative R27K substitution in MVA 183 and the same change is seen in CVA, rabbitpox virus Utrecht (a VACV strain) and VACV strains Acambis 3 and 3737. Second, in B14 *α*-helix 5, there is another conservative substitution (A84V) in MVA. In different VACV strains and other orthopoxviruses, alanine, valine or threonine is found at this position (Fig. 1b). Third, and most notably, there is a 6 aa deletion in MVA 183 corresponding to B14 *α*-helix 6 (*α*6; Fig. 1b), and this deletion is not found in any other VACV strains or orthopoxviruses. The central position of this helix in the B14 structure suggests that this deletion in 183 might affect the overall fold of the protein. In contrast, the other amino acid substitutions are unlikely to do so.

To examine whether protein 183 behaves in an analogous manner to B14 as an inhibitor of NF-*κ*B activation, the *183R* gene including DNA encoding an N-terminal FLAG tag was cloned into the mammalian expression vector pCI (Promega) downstream of a human cytomegalovirus  immediate-early promoter. HEK 293T cells (Chen *et al.*, 2008) were transfected with a reporter plasmid containing firefly luciferase attached to an NF-*κ*B-responsive promoter (Chen *et al.*, 2008), an internal control containing *Renilla* luciferase linked to the thymidine kinase promoter (Chen *et al.*, 2008), and either pCI or pCI encoding FLAG-tagged B14 or 183 (Fig. 2a). After 18 h, the transfected cells were stimulated with tumour necrosis factor alpha (TNF-*α*; Peprotech) for 8 h. NF-*κ*B activation was assessed by firefly luciferase activity and normalized by *Renilla* luciferase activity. Triplicate samples were collected and data are expressed as the mean fold induction relative to pCI. Whilst B14 inhibited NF-*κ*B activation as expected, MVA 183 had no such activity (Fig. 2a). To compare levels of B14 and 183 protein expression, cell lysates from the reporter assays were analysed by immunoblotting with anti-FLAG (Sigma) and anti-*α*-tubulin (Upstate Biotechnology) mAbs (Fig. 2b). B14 was easily detectable, whereas 183 was not. However, after TNF-*α* treatment, the level of 183 increased for unknown reasons. Blotting with an anti-*α*-tubulin mAb confirmed equal loading of samples. It appeared, therefore, that 183 was unstable, but stability was increased by activation of the signalling pathway leading from TNF-*α* to NF-*κ*B activation. Nonetheless, despite 183 expression being detectable after TNF-*α* stimulation, under the conditions tested it was unable to inhibit NF-*κ*B activation.

MVA does not replicate in many mammalian cell lines and is avirulent in mice. Therefore, to investigate the role of MVA 183 in virus virulence, the *183R* gene was inserted into VACV strain WR lacking *B14R*, vΔB14 (Chen *et al.*, 2006), and the virulence of the recombinant virus, vΔB14-MVA183, was measured in a murine i.d. model (Tscharke & Smith, 1999). A pSJH7-based plasmid (Hughes *et al.*, 1991) containing the *183R* open reading frame flanked by 250 bp of WR genomic DNA upstream and downstream of *B14R* was transfected into RK-13 cells (European Collection of Cell Cultures; ECACC) that were infected with vΔB14. A recombinant virus containing MVA 183, vΔB14-MVA183, was selected by transient dominant selection (Falkner & Moss, 1990) and PCR analysis confirmed that vΔB14-MVA183 contained the correct genome structure at the *B14R/183R* locus (see Supplementary Fig. S1, available in JGV Online). vΔB14-MVA183 was purified in parallel with viruses vB14 (wild-type, WR) and vΔB14 (Chen *et al.*, 2006) by sedimentation through a sucrose cushion, and virus infectivity was titrated on BS-C-1 cells (ECACC). Groups of female C57Bl/6 mice, between 6 and 8 weeks old (Harlan), were anaesthetized and inoculated with each virus into each ear pinna (Tscharke & Smith, 1999; Tscharke *et al.*, 2002). Titres of inocula were confirmed by plaque assay. Animals were examined daily and the diameter of lesions at the inoculation site was measured using a micrometer. vΔB14 induced a smaller lesion size than wild-type virus and reinsertion of *B14R* into vΔB14 restored lesion size, as reported previously (Chen *et al.*, 2006). In contrast, insertion of *183R* into vΔB14 did not alter lesion size (Fig. 3a). Therefore, 183 does not contribute to virulence in this model, a conclusion consistent with the protein being unstable and unable to inhibit NF-*κ*B activation in cell culture.

To examine 183 expression in infected cells, lysates from CEFs infected with MVA or vΔB14-MVA183 were analysed by immunoblotting using the anti-B14 polyclonal antibody (Chen *et al.*, 2006) and mouse mAbs against D8 (a VACV late protein) (Parkinson & Smith, 1994) and *α*-tubulin (Fig. 3b). As before, although B14 produced by VACV WR was detected easily, MVA 183 was not detected in cells infected with MVA or vΔB14-MVA183. This difference was unlikely to be due to altered transcription of gene *183R*, as the nucleotide sequence upstream of MVA *183R* was the same in WR *B14R*, and so the difference probably reflected protein instability as noted above (Fig. 2b). A major pathway for the removal of labile proteins in eukaryotic cells is via ubiquitination and degradation by the proteasome. To assess whether protein 183 was being degraded by this mechanism, a proteasome inhibitor (MG132; Satheshkumar *et al.*, 2009) was added to cells prior to and throughout infection. MG132 is a short oligopeptidic sequence with an aldehyde electrophilic structure at the C terminus. It blocks multiple active sites in the proteasome by forming transition-state analogues via interacting with a catalytic hydroxyl/thiol group to form a reversible hemi (thio)acetal. In the presence of MG132, protein 183 expression from MVA or vΔB14-MVA183 was detected and was similar in each case (Fig. 3b). This showed that 183 was labile and degraded by the proteasome. It also indicated that additional factors expressed by VACV WR were unable to stabilize protein 183.

Analysis of VACV protein D8 expression levels in these cells showed a substantial decrease in the presence of MG132. This observation was in accord with the report that MG132 blocks a post-entry step in VACV replication (Satheshkumar *et al.*, 2009). After virus entry and uncoating, viral DNA synthesis is prevented so that virus intermediate- and late-gene expression is inhibited (Satheshkumar *et al.*, 2009). D8 is a late protein and is therefore reduced considerably by MG132. This reduction in D8 expression also served as a control, showing that MG132 was inhibiting proteasome activity.

Several lines of evidence indicate that MVA 183 is a labile protein. First, following transfection of mammalian cells with plasmids encoding 183, the protein was not detected, whereas B14 was. Second, after infection of avian cells by MVA or a VACV WR virus encoding MVA 183, the 183 protein was not detectable unless the proteasome was inhibited by MG132. Third, attempts to express protein 183 in *Escherichia coli* under conditions that yielded stable, soluble B14 protein that was used for protein crystallography (Graham *et al.*, 2008) gave only low yields of insoluble 183 (data not shown). Therefore, 183 is a labile protein and the probable cause is deletion of the REISAI motif in the B14 *α*6 helix. This deletion is found only in MVA and in no other VACV strain, including the parental virus CVA, and so arose during MVA passage in CEFs. The observation that MVA 183 does not inhibit NK-*κ*B activation may explain, at least in part, why MVA infection activates NF-*κ*B in contrast to other VACV strains (Oie & Pickup, 2001). Interestingly, protein 183 was stabilized after treatment of cells with TNF-*α*. Possible explanations for this are alterations to the proteasome-mediated degradation of proteins after TNF-*α* stimulation, or that a component of the TNF-*α*-induced signalling pathway leading to NF-*κ*B activation can somehow stabilize the 183 protein once the pathway is activated. However, if the latter were true, it is notable that, even when 183 is present, at the levels tested it did not inhibit NF-*κ*B activation. It is also formally possible, but less likely, that TNF-*α* treatment is affecting 183 mRNA levels.

The original bioinformatic analysis of the MVA genome recorded open reading frames that were retained, deleted or disrupted by mutation compared with other VACV strains (Antoine *et al.*, 1998), but the functional consequences of smaller mutations, such as those noted here for protein 183, were unknown. Here we demonstrate that, although MVA protein 183 shares 95 % amino acid sequence identity with protein B14 from VACV strain WR, it is unstable and lacks the functions assigned to protein B14, such as inhibition of NF-*κ*B activation and contribution to virus virulence. Other MVA genes encoding immunomodulatory proteins with small deletions or mutations relative to counterparts in other VACV strains are as yet uncharacterized and these changes may have important functional consequences. Attempts to increase the immunogenicity of MVA by engineering or deletion of genes encoding immunomodulators should be informed by knowledge of whether the encoded protein is functional, and priority should be given to engineering genes known to encode functional proteins.

In summary, MVA protein 183 is a non-functional counterpart of VACV WR protein B14 and neither inhibits NF-*κ*B activation following addition of TNF-*α*, nor contributes to VACV strain WR virulence. The loss of function is probably attributable to a 6 aa deletion within the *α*6 helix of the Bcl-2 fold and it is unlikely that removal of this gene from the MVA genome will enhance MVA immunogenicity.

## Supplementary Material

[Supplementary figure]

## Figures and Tables

**Fig. 1.**
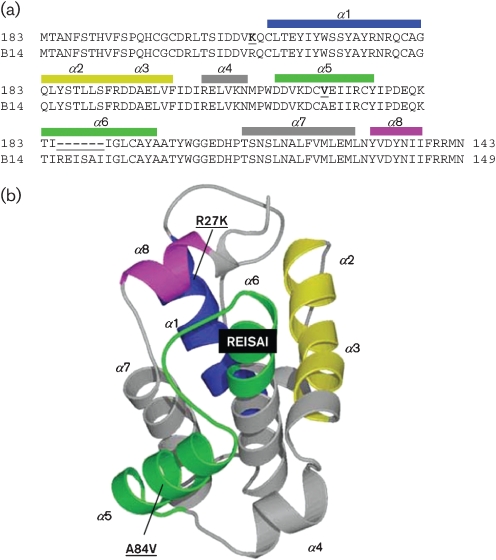
B14 structure. (a) Alignment of proteins WR B14 and MVA 183. Positions of divergence are shown in bold and underlined. The positions of the *α*-helices of the B14 crystal structure are shown above the alignment and are coloured from N to C terminus as shown in the three-dimensional structure in (b). (b) Ribbon representation of the crystal structure of B14. *α*-Helices are marked by sequence from N to C terminus and are coloured from blue (N terminus) to magenta (C terminus). The changes in 183 compared with B14 are shown underlined and in bold, including the REISAI motif that is deleted in 183.

**Fig. 2.**
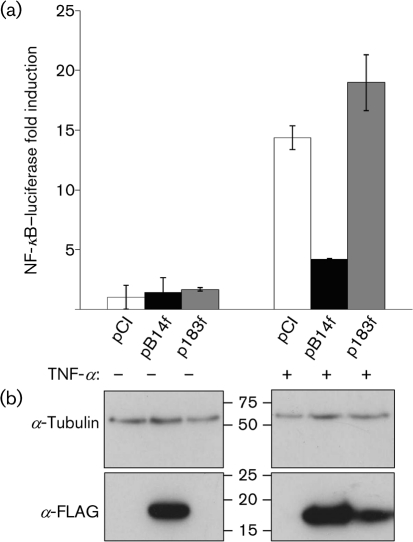
Protein 183 does not inhibit NF-*κ*B activation by TNF-*α*. (a) HEK 293T cells were transfected with either pCI or pCI containing FLAG-tagged B14 or 183 (pB14f and p183f) and a luciferase NF-*κ*B reporter plasmid for 18 h. Cells were then stimulated with 50 ng TNF-*α* ml^−1^ for 8 h and luminescence was assessed as described previously (Chen *et al.*, 2008). The assay was carried out in triplicate and the error bars represent sd from the mean. (b) A small aliquot of the cell lysates was retained and analysed by immunoblotting with anti-FLAG and anti-*α*-tubulin mAbs. The positions of molecular mass markers in kDa are indicated.

**Fig. 3.**
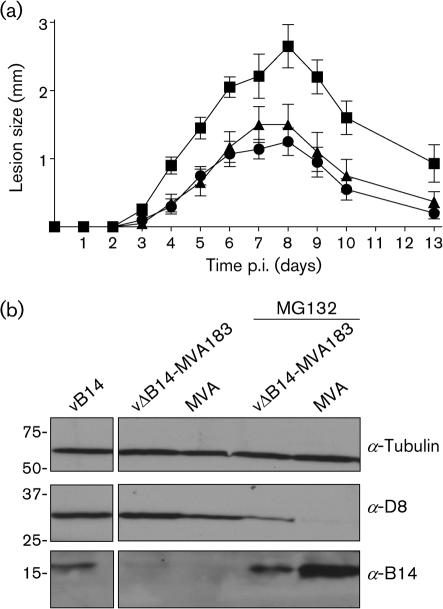
(a) Measurement of virulence of VACV WR strains with or without MVA 183. (a) Groups of eight C57Bl/6 mice were infected i.d. with 10^4^ p.f.u. vB14 (▪), vΔB14 (▴) or vΔB14-MVA183 (•) in 10 μl PBS and the lesion diameter was recorded daily. Error bars represent sem. (b) Expression of B14 and 183 proteins after infection of CEFs. CEFs were infected at 10 p.f.u. per cell with VB14, vΔB14-MVA183 or MVA. Where indicated, cells were treated with MG132 (10 μM) for 1.5 h before and throughout infection. Infected cells were incubated for 8 h before lysis and analysis by SDS-PAGE and immunoblotting with anti-B14, anti-D8 and anti-*α*-tubulin antibodies. The positions of molecular mass markers in kDa are indicated.

## References

[r1] Antoine, G., Scheiflinger, F., Dorner, F. & Falkner, F. G. (1998). The complete genomic sequence of the modified vaccinia Ankara strain: comparison with other orthopoxviruses. Virology 244, 365–396. 9601507 10.1006/viro.1998.9123

[r2] Blanchard, T. J., Alcami, A., Andrea, P. & Smith, G. L. (1998). Modified vaccinia virus Ankara undergoes limited replication in human cells and lacks several immunomodulatory proteins: implications for use as a human vaccine. J Gen Virol 79, 1159–1167. 9603331 10.1099/0022-1317-79-5-1159

[r3] Chen, R. A., Jacobs, N. & Smith, G. L. (2006). Vaccinia virus strain Western Reserve protein B14 is an intracellular virulence factor. J Gen Virol 87, 1451–1458. 16690909 10.1099/vir.0.81736-0

[r4] Chen, R. A., Ryzhakov, G., Cooray, S., Randow, F. & Smith, G. L. (2008). Inhibition of I*κ*B kinase by vaccinia virus virulence factor B14. PLoS Pathog 4, e22 18266467 10.1371/journal.ppat.0040022 PMC2233672

[r5] Clark, R. H., Kenyon, J. C., Bartlett, N. W., Tscharke, D. C. & Smith, G. L. (2006). Deletion of gene *A41L* enhances vaccinia virus immunogenicity and vaccine efficacy. J Gen Virol 87, 29–38. 16361415 10.1099/vir.0.81417-0

[r6] Falkner, F. G. & Moss, B. (1990). Transient dominant selection of recombinant vaccinia viruses. J Virol 64, 3108–3111. 2159565 10.1128/jvi.64.6.3108-3111.1990 PMC249504

[r7] Fenner, F., Anderson, D. A., Arita, I., Jezek, Z. & Ladnyi, I. D. (1988). *Smallpox and its Eradication*. Geneva: World Health Organization.

[r8] Graham, S. C., Bahar, M. W., Cooray, S., Chen, R. A., Whalen, D. M., Abrescia, N. G., Alderton, D., Owens, R. J., Stuart, D. I. & other authors (2008). Vaccinia virus proteins A52 and B14 share a Bcl-2-like fold but have evolved to inhibit NF-*κ*B rather than apoptosis. PLoS Pathog 4, e1000128 18704168 10.1371/journal.ppat.1000128 PMC2494871

[r9] Hashizume, S., Yoshizawa, H., Morita, M. & Suzuki, K. (1985). Properties of attenuated mutant of vaccinia virus, LC16m8, derived from Lister strain. In *Vaccinia Viruses as Vectors for Vaccine Antigens*, pp. 87–99. Edited by G. V. Quinnan. New York: Elsevier Science.

[r10] Hughes, S. J., Johnston, L. H., de Carlos, A. & Smith, G. L. (1991). Vaccinia virus encodes an active thymidylate kinase that complements a *cdc8* mutant of *Saccharomyces cerevisiae*. J Biol Chem 266, 20103–20109. 1657913

[r11] Lane, J. M., Ruben, F. L., Neff, J. M. & Millar, J. D. (1969). Complications of smallpox vaccination, 1968. National surveillance in the United States. N Engl J Med 281, 1201–1208. 4186802 10.1056/NEJM196911272812201

[r12] Mahnel, H. & Mayr, A. (1994). Experiences with immunization against orthopox viruses of humans and animals using vaccine strain MVA. Berl Munch Tierarztl Wochenschr 107, 253–256 (in German). 7945180

[r13] Mayr, A. & Munz, E. (1964). Changes in the vaccinia virus through continuing passages in chick embryo fibroblast cultures. Zentralbl Bakteriol Orig 195, 24–35 (in German). 5890664

[r14] Mayr, A., Stickl, H., Muller, H. K., Danner, K. & Singer, H. (1978). The smallpox vaccination strain MVA: marker, genetic structure, experience gained with the parenteral vaccination and behavior in organisms with a debilitated defence mechanism. Zentralbl Bakteriol [B] 167, 375–390 (in German). 219640

[r15] Meisinger-Henschel, C., Schmidt, M., Lukassen, S., Linke, B., Krause, L., Konietzny, S., Goesmann, A., Howley, P., Chaplin, P. & other authors (2007). Genomic sequence of chorioallantois vaccinia virus Ankara, the ancestor of modified vaccinia virus Ankara. J Gen Virol 88, 3249–3259. 18024893 10.1099/vir.0.83156-0

[r16] Meyer, H., Sutter, G. & Mayr, A. (1991). Mapping of deletions in the genome of the highly attenuated vaccinia virus MVA and their influence on virulence. J Gen Virol 72, 1031–1038. 2033387 10.1099/0022-1317-72-5-1031

[r17] Moss, B. (2007). *Poxviridae*: the viruses and their replicaton. In *Fields Virology*, 5th edn, pp. 2905–2946. Edited by D. M. Knipe & P. M. Howley. Philadelphia, PA: Lippincott Williams & Wilkins.

[r18] Oie, K. L. & Pickup, D. J. (2001). Cowpox virus and other members of the orthopoxvirus genus interfere with the regulation of NF-*κ*B activation. Virology 288, 175–187. 11543670 10.1006/viro.2001.1090

[r19] Parkinson, J. E. & Smith, G. L. (1994). Vaccinia virus gene A36R encodes a *M*_r_ 43–50 K protein on the surface of extracellular enveloped virus. Virology 204, 376–390. 8091668 10.1006/viro.1994.1542

[r20] Satheshkumar, P. S., Anton, L. C., Sanz, P. & Moss, B. (2009). Inhibition of the ubiquitin–proteasome system prevents vaccinia virus DNA replication and expression of intermediate and late genes. J Virol 83, 2469–2479. 19129442 10.1128/JVI.01986-08 PMC2648259

[r21] Smith, G. L., Mackett, M. & Moss, B. (1983). Infectious vaccinia virus recombinants that express hepatitis B virus surface antigen. Nature 302, 490–495. 6835382 10.1038/302490a0

[r22] Staib, C., Kisling, S., Erfle, V. & Sutter, G. (2005). Inactivation of the viral interleukin 1*β* receptor improves CD8^+^ T-cell memory responses elicited upon immunization with modified vaccinia virus Ankara. J Gen Virol 86, 1997–2006. 15958679 10.1099/vir.0.80646-0

[r23] Stickl, H., Hochstein-Mintzel, V., Mayr, A., Huber, H. C., Schafer, H. & Holzner, A. (1974). MVA vaccination against smallpox: clinical tests with an attenuated live vaccinia virus strain (MVA). Dtsch Med Wochenschr 99, 2386–2392 (in German). 4426258 10.1055/s-0028-1108143

[r24] Sutter, G. & Moss, B. (1992). Nonreplicating vaccinia vector efficiently expresses recombinant genes. Proc Natl Acad Sci U S A 89, 10847–10851. 1438287 10.1073/pnas.89.22.10847 PMC50439

[r25] Tscharke, D. C. & Smith, G. L. (1999). A model for vaccinia virus pathogenesis and immunity based on intradermal injection of mouse ear pinnae. J Gen Virol 80, 2751–2755. 10573171 10.1099/0022-1317-80-10-2751

[r26] Tscharke, D. C., Reading, P. C. & Smith, G. L. (2002). Dermal infection with vaccinia virus reveals roles for virus proteins not seen using other inoculation routes. J Gen Virol 83, 1977–1986. 12124461 10.1099/0022-1317-83-8-1977

