# Supplementary material for: Mutations in modified virus Ankara protein 183 render it a non-functional counterpart of B14, an inhibitor of nuclear factor κB activation
Source: J Gen Virol. 2010 Sep;91(Pt 9):2216–20. doi: 10.1099/vir.0.022343-0 (PMC3052518; doi:10.1099/vir.0.022343-0)
Supplement: [Supplementary figure] [file supp_91_9_2216__index.html]

 Mutations in modified virus Ankara protein 183 render it a non-functional counterpart of B14, an inhibitor of nuclear factor {kappa}B activation -- McCoy et al. 91 (9): 2216 Data Supplement - Supplementary figure -- Journal of General Virology

### Mutations in modified virus Ankara protein 183 render it a non-functional counterpart of B14, an inhibitor of nuclear factor κB activation, by L. E. McCoy, A. S. Fahy, R. A.-J. Chen and G. L. Smith

*Journal of General Virology* vol. **91**, part 9, pp. 2216–2220

  

**Supplementary Fig. S1.** PCR analysis of the *B14R* locus [PDF] (75 KB)

  
  
