# Supplementary material for: Mutations in modified virus Ankara protein 183 render it a non-functional counterpart of B14, an inhibitor of nuclear factor κB activation
Source: J Gen Virol. 2010 Sep;91(Pt 9):2216–20. doi: 10.1099/vir.0.022343-0 (PMC3052518; doi:10.1099/vir.0.022343-0)
Supplement: [Supplementary figure] [file supp_91_9_2216__1.pdf]

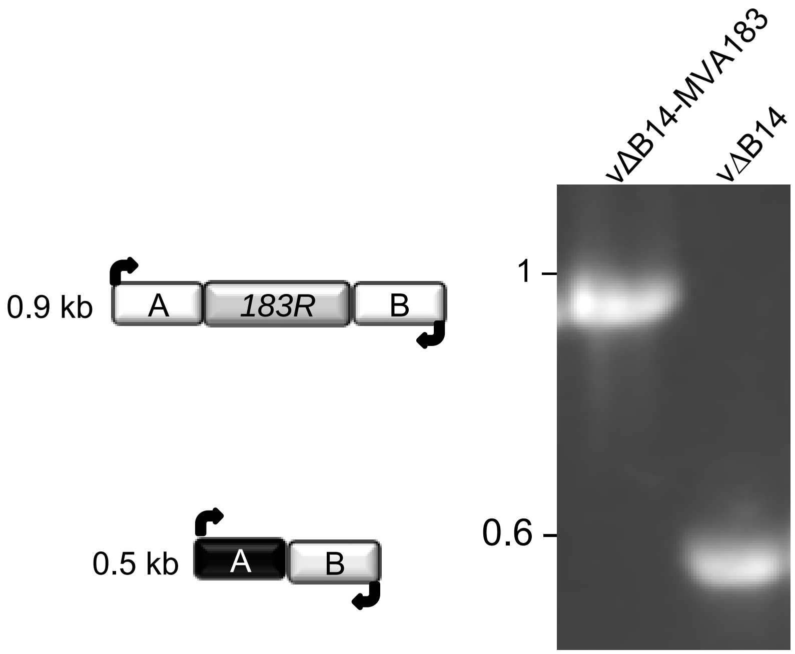

Supplementary Fig. S1. PCR analysis of the *B14R* locus. DNA was extracted from cells infected with vΔB14 or vΔB14-MVA 183 and used as template for a PCR using oligonucleotide primers FAf (5′-GGAATTCCTTCGGTTCAACTGGAGATTA) and FBr (5′-GCTCTAGAGCATTGCTACCATTATCTACGG) that anneal to the indicated positions flanking the *B14R* gene. PCR products were analysed by agarose gel electrophoresis. A PCR product of approximately 0.9 kb was produced from the vΔB14-MVA 183 virus whereas a product of 0.5 kb was produced with vΔB14 DNA as template. The positions of dsDNA size markers are indicated.

McCoy, L. E., Fahy, A. S., Chen, R. A.-J. & Smith, G. L. (2010). Mutations in modified virus Ankara protein 183 render it a non-functional counterpart of B14, an inhibitor of nuclear factor κB activation. *J Gen Virol* **91**, 2216–2220.
